# Income-based inequalities in caregiving time and depressive symptoms among older family caregivers under the Japanese long-term care insurance system: A cross-sectional analysis

**DOI:** 10.1371/journal.pone.0194919

**Published:** 2018-03-28

**Authors:** Tami Saito, Naoki Kondo, Koichiro Shiba, Chiyoe Murata, Katsunori Kondo

**Affiliations:** 1 Department of Social Science, National Center for Geriatrics and Gerontology, Obu, Japan; 2 Department of Health and Social Behavior, School of Public Health, The University of Tokyo, Tokyo, Japan; 3 Department of Social and Behavioral Sciences, Harvard T.H. Chan School of Public Health, Boston, Massachusetts, United States of America; 4 Center for Preventive Medical Science, Chiba University, Chiba, Japan; 5 Department of Gerontological Evaluation, National Center for Geriatrics and Gerontology, Obu, Japan; University of West London, UNITED KINGDOM

## Abstract

**Aim:**

Long-term care systems may alleviate caregiver burdens, particularly for those with fewer resources. However, it remains unclear whether socioeconomic disparity in caregiver burdens exists under a public, universal long-term care insurance (LTCI) system. This study examined income-based inequalities in caregiving time and depressive symptoms in Japanese older family caregivers. We further compared inequality in depressive symptoms with that of non-caregivers to evaluate whether family caregiving exacerbates this disparity.

**Methods:**

Data were obtained from a cross-sectional, nationwide survey conducted by the Japan Gerontological Evaluation Study in 2013. Participants were functionally independent older adults aged ≥65 years (*N* = 21,584). Depressive symptoms were assessed using the Geriatrics Depression Scale (GDS); caregiving hours per week, household income, and other covariates were also assessed.

**Results:**

Family caregivers occupied 8.3% of the total. A Poisson regression model revealed that caregivers in lower income groups (compared to those in the highest) were 1.32 to 1.95 and 1.63 to 2.68 times more likely to engage in ≥36 and ≥72 hours/week of caregiving, respectively. As for the GDS (≥5), an excess risk was found in the caregivers in lower (compared to higher) income groups (adjusted prevalence ratio: 1.57–3.10). However, an interaction effect of income by caregiving role indicated no significant difference in inequality between caregivers and non-caregivers (*p* = .603). The excess risk for GDS (≥5) in the caregivers compared to non-caregivers was observed across income groups.

**Conclusions:**

Our findings revealed a possible disparity in family caregivers under the public LTCI system. Further studies should examine factors associated with longer caregiving hours in lower income households. Our findings also suggest the necessity for more efforts to alleviate depressive symptoms in family caregivers under the LTCI system regardless of income level, rather than exclusively supporting those with a low income.

## Introduction

As the number of older adults requiring care increases globally [1], caregiving could impact more people’s lives (i.e., typically family members’ lives). Family caregiving is associated with depression [2, 3], hypertension [4], chronic fatigue [5], undesirable health behaviors [6], and withdrawal from the workforce [7, 8]. Particularly, depression is a leading cause of global disease burden [9]. Therefore, alleviating caregiver depression is a critical worldwide public health issue.

Several models, such as the stress-process model [10], stress-appraisal model [11], and their applied models [12], are known to have attempted successfully to explain family caregivers’ depression through a process of family caregiving experiences. According to these models, caregivers’ depression is caused by an interaction between stressors (e.g., care recipients’ physical and cognitive impairment), appraisals for the stressors (e.g., caregiving time, overload, or others depending on the models), and resources (e.g., social support for and coping skills of caregivers). The stress-process model further suggests that these elements differ by family caregivers’ backgrounds such as socioeconomic status (SES) or availability for services [10]. Caregivers with higher SES could afford more services they require, and have more skills, knowledges, and other socio-cultural and psychological resources to cope efficiently with the stressors [13, 14]. Studies have suggested that caregivers with lower SES experienced increased depression [15], caregiving burden [16], and hours spent on caregiving [17], as well as decreased access to, and use of, formal services [18, 19]. However, few studies have compared caregivers with non-caregivers; consequently, it remains unclear whether the inequality is exacerbated by caregiving itself in addition to other disadvantages that caregivers with lower SES may experience in their daily lives.

The World Health Organization has recommended that all countries develop a long-term care system that provide affordable and accessible services to older adults who require them, particularly for those with less access to resources [1]. For instance, Japan implemented a public, mandatory, and universal long-term care insurance (LTCI) system in 2000 to socialize care that has been traditionally based on familism [20]. People aged 65 years and over requiring care are eligible for care-related in-kind benefits regardless of income and care availability [21]. As of 2013, users could purchase services at 10% of the co-insurance payment. Even under this relatively egalitarian system [22], several studies suggested socioeconomic inequality in depressive symptoms [2] and service utilization among caregivers [23, 24]. However, few studies have examined the inequality in caregiving time [25], and, to the best of our knowledge, no study in Japan has examined social disparity in depressive symptoms among family caregivers compared to those among non-caregivers.

Therefore, the current cross-sectional study examined socioeconomic disparity in depressive symptoms (regarded as a consequence of caregiver burden) and caregiving time (an objective measure of caregiver burden) [17, 26, 27] among Japanese older family caregivers. We focused on income among a variety of socioeconomic indicators, because it could be the most likely factor to reflect physical access to care services and information. We hypothesized that, due to the lower access to those resources, family caregivers with a lower income were more likely to engage in longer hours of caregiving and more likely to have depressive symptoms. In addition, to elucidate whether the observed income-based inequality was directly related to the caregiving itself, we compared income-based disparity in depressive symptoms between family caregivers and non-caregivers. We thought that if the income-based inequality was attributable to caregiving, the disparity in depressive symptoms would be larger among caregivers than non-caregivers.

## Methods

### Participants and data collection

Data targeting a relatively healthy older population, who were potential family caregivers or non-caregivers, but rarely care recipients, were obtained from the Japan Gerontological Evaluation Study (JAGES). The primary purpose of this national project was to examine the social determinants of health among functionally independent older adults, aged ≥65 years [28]. In this study, data from the 2013 wave were used because the data were the most recent among those enabling us to examine our study aim. JAGES 2013 respondents included 193,694 older adults across 30 municipalities in Japan. Respondents were selected using complete enumeration (for 13 smaller-scale municipalities) or random sampling (for 17 larger-scale municipalities). Although the selection of municipalities was not random, they included both rural and urban areas and covered most regions of Japan. No participants were eligible for LTCI benefits, implying that they had no functional disability.

A mailed, self-administered, questionnaire survey was administered between October and December 2013, and 137,736 valid responses were obtained (valid response rate: 71.1%). The JAGES questionnaire consists of basic questions to be completed by all respondents, as well as five separate modules that are randomly allocated to participants (20% probability for each module). In this study, we utilized Module C, which included items related to caregiving status. This study’s protocol and informed consent procedure were approved by the Nihon Fukushi University Ethics Committee (No. 13–14). The survey was implemented under a research agreement between each municipality and the JAGES project. The municipalities conducted the survey and provided anonymous data to us, the JAGES researchers. We assumed voluntary response as consent to participate. Of the 25,927 respondents for Module C, we analyzed data from 21,584 respondents who provided information on caregiving status.

### Measurements

#### Depressive symptoms

Depressive symptoms were assessed using a 15-item Japanese version of the Geriatric Depression Scale (GDS) [29], which has confirmed reliability and validity among community-dwelling older Japanese adults [30]. Participants respond to the GDS items using a simple “*yes*” or “*no*” format. Scores ranged from 0 to 15; higher scores reflected increased depressive symptoms. We dichotomized the score as a cut-off of ≥5 points [31], which indicates the presence of mild to severe depressive symptoms.

#### Caregiving hours

We assessed hours of caregiving per week, using caregiving frequency and hours of caregiving per caregiving day [32]. Frequency of caregiving was assessed using the options “*almost every day*,” “*two to four days a week*,” “*once a week*,” and “*one to three times a month*.” Hours of caregiving per caregiving day were evaluated using five options: “*almost all day*,” “*almost half a day*,” “*several hours per day*,” “*providing help if necessary*,” and “*other*.” To calculate frequency of caregiving hours per week, we operationally categorized “*almost every day*” as 6, “*two to four days*” as 3, and “*one to three times a month*” as 0.5 days per week. Regarding caregiving hours per week, “*almost all day*” was scored as 12 hours, “*almost half a day*” as 6, “*several hours*” as 2.5, and “*providing help if necessary*” as 1 [2,33]. These calculations resulted in caregiving hours per week score ranging from 0.5 to 72 hours. Due to its skewed distribution, we dichotomized caregiving hours according to a study suggesting increased depression among caregivers engaging in ≥36 hours of care per week [34]. This cut-off point is nearly equivalent to legal full-time employment hours in Japan [35]. We also examined ≥72 hours per week to elucidate the relationship between income level and the extremely long caregiving hours.

#### Caregiving role

Caregiving role was assessed with one item that asked whether the respondent provides care for a frail family member. The item included “*not providing care*;” “*mainly providing care*;” and *“not mainly providing* care, *but supporting primary caregiver in relation to care*” as options. We regarded the latter two categories as caregivers.

#### Household income

Annual household income (Japanese yen; JPY) was equivalized to adjust for the number of family members in a household, and categorized into quartiles (1st quartile: 3,180,000 JPY or higher; 2nd quartile: between 2,000,000 and 3,179,999 JPY; 3rd quartile: between 1,300,000 and 1,999,999 JPY; and 4th quartile: 1,299,999 JPY or lower). The 4th quartile nearly equaled the Japanese poverty line during the survey period (i.e., 1,220,000 JPY) [32]. In addition, respondents receiving public assistance were independently categorized because they had co-insurance and monthly premium payment exemptions, while others had paid 10% co-insurance during the survey period. Public assistance services in Japan are available for a person whose standard of living is lower than a minimum level. We also included a “missing” category. JPY equaled approximately 0.01 US dollars during the survey period.

#### Covariates

Among the potential confounders in the relationship between income and the outcomes, we assessed the presence of diseases or symptoms (*none/one/two or more*), marital status (*married/not married*), work engagement (*yes/no*), education *(<10/≥10 years*), age (*in years*), and gender. Except for age and gender, all covariates included a category of “missing.”

### Statistical analyses

The outcomes of depression and long caregiving hours were relatively common (10% or higher); therefore, general logistic regression models could overestimate the risk ratios of the outcomes across income groups. Instead, we used a Poisson regression model, as suggested by Zhang and Yu [36].

First, we estimated adjusted prevalence ratios (APRs) of long hours of caregiving (≥36 hours and ≥72 hours) in each income group, compared to the highest income group as the reference, controlling for age, gender, education, marital status, work engagement, and disease status. Similarly, we estimated APRs of the presence of depressive symptoms (GDS scored ≥5) in each income group using the same model. To compare the income-based inequality in the depressive symptoms with those in the non-caregivers, we estimated the APRs using the non-caregiver sample, and estimated the interaction effect of income by caregiving role within the same model using the whole sample. According to this interaction, we also estimated the APRs of the GDS (≥5) in the caregivers compared to the non-caregivers, stratified by the income groups, to elucidate the relative risk of depressive symptoms based on caregiving role across income levels.

All analyses were conducted using SPSS version 24.0J (IBM Japan, Tokyo, Japan). Statistical significance was set at *p* < .05.

### Results

Participants’ mean age was 73.8 years (age range = 65 to 99 years) and 47.7% were men. Further, 1,782 (8.3%) respondents engaged in caregiving, with 51.9% of these providing care for a spouse, 23.7% for a parent, 11.8% for a parent-in-law, and 12.5% for another family member. Among the caregivers, 25.7% and 16.7% engaged in ≥36 and ≥72 hours of caregiving per week, respectively.

Table 1 shows caregivers’ characteristics compared to those of non-caregivers. Compared to non-caregivers, caregivers were younger, more likely to be a woman, more likely to be married, and had ≥10 years of education. Caregivers were more likely to have depressive symptoms (GDS ≥5) than non-caregivers (26.4% vs 21.1%, respectively).

**Table 1:** Characteristics of caregivers compared to non-caregivers.

Variables	Caregivers (*n* = 1,782)	Non-caregivers (*n* = 19,802)	
	*M* (SD)^a^ or %	*M* (SD)^a^ or %	*p*^b^
**Age (range: 65–99 years)**	73.2 (6.2)	73.8 (6.1)	< .001
**Gender (male)**	44.4	48.0	.001
**Income** ^c^			.034
1st quartile	18.5	19.8	
2nd quartile	23.1	22.3	
3rd quartile	19.4	19.9	
4th quartile	24.2	21.3	
Public assistance	1.2	1.5	
Missing	13.6	15.1	
**Education**			.001
<10 years	35.0	40.2	
≥10 years	63.4	58.3	
Missing	1.7	1.6	
**Marital status**			.001
Married	86.1	70.9	
Not married	10.4	26.7	
Missing	3.4	2.4	
**Work engagement**			.039
Yes	20.4	22.9	
No	74.2	71.4	
Missing	5.3	5.6	
**Presence of disease**			.292
None	14.9	14.5	
One	35.1	33.6	
Two or more	43.4	45.8	
Missing	6.5	6.2	
**GDS**^d^			< .001
<5	56.0	63.0	
≥5	26.4	21.1	
Missing	17.6	16.0	

^a^M: mean; SD: standard deviation.

^b^Differences in distributions between caregivers and non-caregivers were assessed using chi-squared tests. Difference in mean age were compared using t-tests.

^c^1st quartile: 3,180,000 JPY or higher; 2nd quartile: between 2,000,000 and 3,179,999 JPY; 3rd quartile: between 1,300,000 and 1,999,999 JPY; and 4th quartile: 1,299,999 JPY or lower. Public assistance group was independently categorized.

^d^Geriatric Depression Scale.

Table 2 shows the characteristics of the caregivers with or without depressive symptoms (GDS ≥5) or ≥36 hours of caregiving. The respondents with depressive symptoms (GDS ≥5) were older, had lower incomes, and were more likely to engage in ≥36 hours of caregiving. Those engaging in ≥36 hours of caregiving were older, more likely to be female, had lower incomes, and were less likely to work. The characteristics of caregivers engaging ≥72 hours of caregiving are shown in S1 Table.

**Table 2:** Characteristics of caregivers according to geriatric depression scale score (≥5) and engagement in long caregiving hours (≥36).

Variables	GDS^a^ (<5) (*n* = 998)	GDS^a^ (≥5) (*n* = 471)		<36 hours (*n* = 1,140)	≥36 hours (*n* = 458)	
	*M* (SD)^b^ or %	*M* (SD)^b^ or %	*p*^c^	*M* (SD)^b^ or %	*M* (SD)^b^ or %	*p*^c^
**Age (range: 65–98 years)**	72.5 (5.9)	73.7 (6.5)	.001	72.6 (6.1)	74.3 (6.2)	< .001
**Gender (Male)**	46.1	49.9	.179	47.8	34.7	< .001
**Income**^d^			< .001			.009
1st quartile	24.8	9.6		20.8	13.5	
2nd quartile	27.1	19.1		23.6	24.0	
3rd quartile	17.5	24.0		20.2	20.3	
4th quartile	19.4	32.7		22.5	27.1	
Public assistance	0.6	1.7		0.8	1.7	
Missing	10.5	13.0		12.2	13.3	
**Education**			< .001			.009
<10 years	29.4	40.6		31.8	39.1	
≥10 years	69.6	57.7		66.9	59.0	
Missing	1.0	1.7		1.2	2.0	
**Marital status**			.198			.639
Married	87.2	86.2		86.2	87.3	
Not married	10.6	10.0		10.9	9.4	
Missing	2.2	3.8		2.9	3.3	
**Work engagement**			.001			< .001
Yes	23.9	15.7		22.9	12.2	
No	73.0	79.4		73.4	81.2	
Missing	3.0	4.9		3.7	6.6	
**Presence of disease**			< .001			.214
None	18.3	10.6		16.0	12.0	
One	35.5	29.9		32.6	33.2	
Two or more	40.7	54.6		45.7	48.0	
Missing	5.5	4.9		5.7	6.8	
**CG hours**^e^			< .001	-	-	-
<36	68.7	59.9		-	-	-
≥36	21.7	31.4		-	-	-
Missing	9.5	8.7		-	-	-

^a^GDS: Geriatric Depression Scale.

^b^M: mean; SD: standard deviation.

^c^Differences in distributions between caregivers with or without GDS (≥5) and engagement in ≥36 caregiving hours were assessed using chi-squared tests. Difference in mean age was compared using a t-test.

^d^1st quartile: 3,180,000 JPY or higher; 2nd quartile: between 2,000,000 and 3,179,999 JPY; 3rd quartile: between 1,300,000 and 1,999,999 JPY; and 4th quartile: 1,299,999 JPY or lower. Public assistance group was independently categorized.

^e^Caregiving hours per week.

Table 3 shows the association between ≥36 or ≥72 hours of caregiving and income levels. Compared to the highest income group, the lower income groups were from 1.32 to 1.95 times more likely to engage in ≥36 hours of caregiving, even after adjusting for covariates. Similarly, those groups were from 1.63 to 2.68 times more likely to engage in ≥72 hours of caregiving.

**Table 3:** Adjusted prevalence ratios (95% confidence intervals) for engaging in long caregiving hours (≥36 or ≥72) by income group^a^.

Variables	≥36 hours	≥72 hours
	*APR (CI)*^b^^c^	*APR (CI)*^b^^c^
**Income**^d^ **(ref: 1st quartile)**		
2nd quartile	1.38 (1.01, 1.88)	1.63 (1.08, 2.46)
3rd quartile	1.32 (0.96, 1.83)	1.86 (1.23, 2.81)
4th quartile	1.43 (1.05, 1.96)	1.79 (1.19, 2.69)
Public assistance	1.95 (0.93, 4.12)	2.68 (1.11, 6.45)
Missing	1.19 (0.83, 1.71)	1.62 (1.03, 2.55)

^a^A Poisson regression model was utilized for data analyses (n = 1598).

^b^Age, gender, education, marital status, work engagement, and disease were adjusted.

^c^APR: adjusted prevalence ratio; CI: 95% confidence interval.

^d^1st quartile: 3,180,000 JPY or higher; 2nd quartile: between 2,000,000 and 3,179,999 JPY; 3rd quartile: between 1,300,000 and 1,999,999 JPY; and 4th quartile: 1,299,999 JPY or lower. Public assistance group was independently categorized.

Table 4 shows the association of income levels and depressive symptoms (GDS ≥5) among caregivers compared to non-caregivers. The characteristics of non-caregivers with depressive symptoms (GDS ≥5) are shown in S2 Table. In both groups, the lower income groups were more likely to have depressive symptoms (GDS ≥5); the public assistance group in the caregivers and non-caregivers were 3.10 and 2.70 times more likely to have depressive symptoms (GDS ≥5) compared to the highest income group, respectively. Although the ARPs in each lower income group was higher in the caregivers than in those in the non-caregivers, the interaction effect of the income levels by caregiving role was not significant (global test p = .603), indicating no larger association in the caregivers compared to that among non-caregivers.

**Table 4:** Adjusted prevalence ratios (95% confidence intervals) for geriatric depression scale score (≥5) in caregivers and non-caregivers by income group^a^.

Variables	CG^b^ (n = 1,469)	Non-CG^b^ (*n* = 16,641)	
	*APR (CI)*^c^^d^	*APR (CI)*^c^^d^	*Interaction p*^e^
**Income**^f^ **(ref: 1st quartile)**			.603
2nd quartile	1.57 (1.09, 2.24)	1.48 (1.32, 1.66)	
3rd quartile	2.32 (1.63, 3.28)	1.76 (1.57, 1.97)	
4th quartile	2.62 (1.87, 3.68)	2.30 (2.07, 2.56)	
Public assistance	3.10 (1.45, 6.63)	2.70 (2.19, 3.32)	
Missing	2.18 (1.47, 3.24)	1.77 (1.57, 2.01)	

^a^A Poisson regression model was utilized for data analysis.

^b^CG: caregivers

^c^Age, gender, education, marital status, work engagement, and disease were adjusted.

^d^APR: adjusted prevalence ratios; CI: 95% confidence intervals.

^e^Interaction term of income by caregiving role was estimated using total sample (CG + non-CG). Age, gender, education, marital status, work engagement, disease, and caregiving role were adjusted. P-value for global test is shown.

^f^1st quartile: 3,180,000 JPY or higher; 2nd quartile: between 2,000,000 and 3,179,999 JPY; 3rd quartile: between 1,300,000 and 1,999,999 JPY; and 4th quartile: 1,299,999 JPY or lower. Public assistance group was independently categorized.

**Fig 1:**
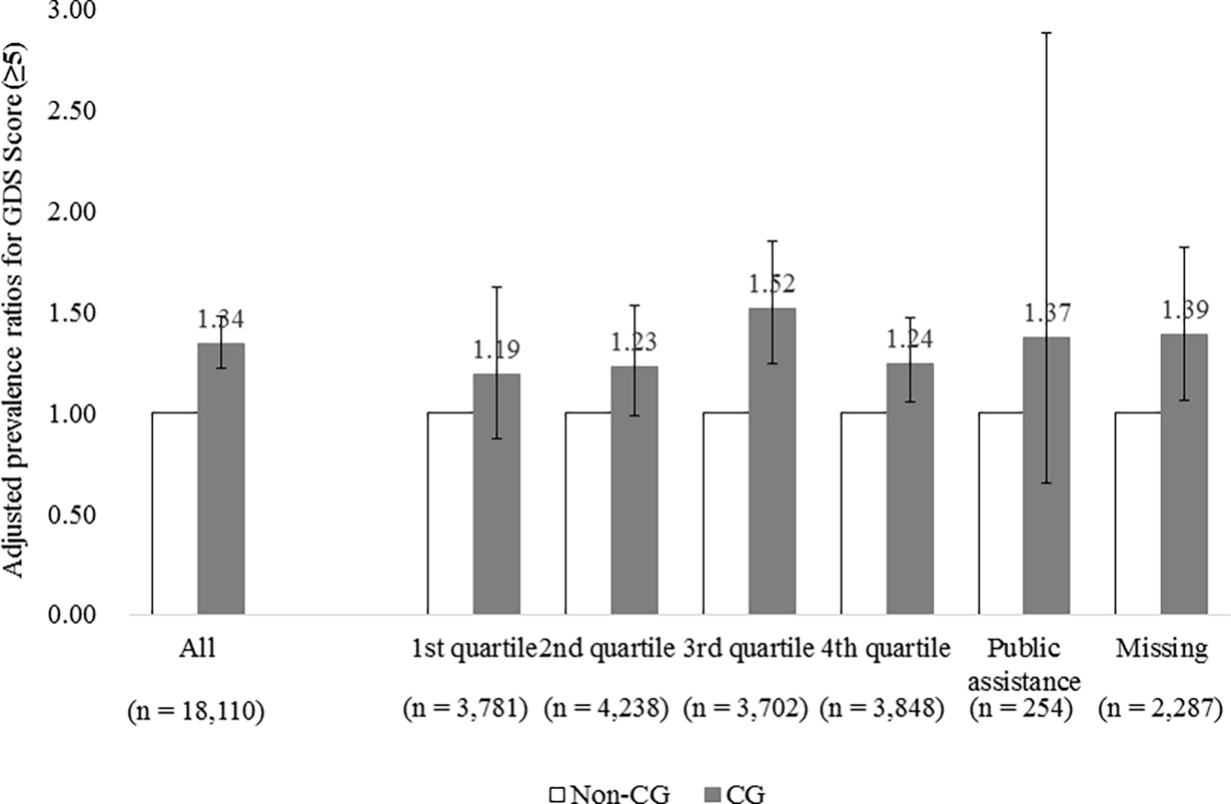
Adjusted prevalence ratios (95% confidence intervals) for geriatric depression scale score (≥5) in caregivers compared to those of non-caregivers stratified by income group. Note: GDS: Geriatric Depression Scale; CG: caregivers.

We also plotted the interaction of income and caregiving role by showing the APRs of depressive symptoms (GDS ≥5) of caregivers compared to those of non-caregivers stratified by income groups (Fig 1). Fig 1 shows that the caregivers were 1.19 to 1.52 times more likely to have depressive symptoms (GDS ≥5) compared to the non-caregivers. However, the APRs did not increase linearly in the lower income groups (trend *p* = .508). The APR in the 3rd income group was the highest among the stratum.

Finally, a series of sensitivity analysis was conducted. First, we examined the difference in caregiving hours and depressive symptoms between the income groups, limiting the caregiver respondents to the primary caregivers (*n* = 533) as selected in a previous study [2]. Compared to the highest income group, the excess risk for ≥36 hours and >72 hours of caregiving in the lower income groups ranged from 1.06 to 1.20 and 1.48 to 1.89, respectively, which were smaller than those of the total caregiver sample. As for depressive symptoms (GDS ≥5), the excess risk of the lower income groups was 1.35 to 2.64, which were relatively similar to those using the total caregivers. Second, the association between income levels and GDS was examined, using a cut-off of 10 points, indicating more severe levels of depressive symptoms. This analysis showed that the interaction term for caregiving by income was similarly non-significant (*p* = .565) to the original analysis using a cut-off of 5.

## Discussion

This study’s findings demonstrated that family caregivers with a lower income (compared to a higher income) were more likely to engage in long caregiving hours, even under a public, universal LTCI system of Japan. Similarly, the caregivers with a lower income (compared to a higher income) were more likely to have depressive symptoms; however, the socioeconomic disparity were comparable to those among non-caregivers.

Regarding the association between income and caregiving hours, our findings were consistent with previous studies [17, 37]; in addition, although few studies have examined the association between SES and caregiving hours in Japan, our finding was consistent with a previous study [25].

Contrary to the German LTCI [38], benefits from the Japanese LTCI are limited to in-kind services, implying that it is not very possible that family caregivers with a lower income provide longer hours of caregiving because of preferring more cash benefits to the in-kind. Instead, it could be speculated that LTCI and other formal service expenses may act as a barrier to meeting support needs of caregivers with a lower income as observed in other countries [18, 19]. Although LTCI recipients in Japan from very low-income households could be partially exempted from out-of-pocket expenditures, the exemption is only available if recipients apply for it from insurers. Therefore, it is possible that a portion of caregivers from such households did not apply for the exemption due to a lack of information or stigma, and restrain utilizing full amount of LTCI services. However, our study also showed that the caregivers receiving public assistance were more likely to engage in longer caregiving hours, despite their exempted co-insurance [23]. Therefore, further studies should carefully examine the detailed factors associated with longer caregiving hours in these households.

Our findings also showed a disparity in depressive symptoms in family caregivers. This is consistent with previous studies in Japan [2] and other countries [15]. However, we failed to detect a synergy effect of income by caregiving role on depressive symptoms. This implies that the observed disparity in caregivers may be caused by other hardships rather than by caregiving itself. Another possibility is that the caregivers with a lower income are more resilient to caregiving stress [37], such as long caregiving hours. Consequently, our findings suggest no evidence for the necessity to develop a program exclusively for caregivers with a lower income without coping with disparity in the whole older population.

Instead, the findings of this study showed that caregivers were more likely to have depressive symptoms, even in the high-income groups. This suggests the necessity for more intensive support toward alleviating depressive symptoms in family caregivers, regardless of the income level.

### Strengths and limitations

Our study has strengths and limitations. We revealed income-based inequality in family caregivers under a public, universal LTCI system. Moreover, our study compared the disparity with that among non-caregivers to elucidate whether the disparity was associated with caregiving itself. Another strength is that our study sample used data from relatively healthy older adults. This could decrease selection bias—that caregivers are spuriously healthier than non-caregivers because unhealthy older adults in general cannot provide care to others. We also used a large dataset, including over 21,000 respondents to a nationwide survey in Japan.

Concurrently, several limitations to this study should be noted. First, the use of a cross-sectional design did not allow for conclusions about causal relationships between income and the outcomes. Likewise, causal relationships between caregiving and depressive symptoms could not be definitively established. Therefore, future studies should utilize a longitudinal study design that could capture these changes and allow for increased causal inferences. Second, the generalizability of this study’s findings should be considered. Our study used data from community-dwelling older adults. Therefore, the findings cannot be applied to middle-aged or younger caregivers. In addition, a moderate response rate of about 70% implies that it is possible that partial caregivers did not participate in the survey because of very intensive caregiving. Third, depressive symptoms and caregiving hours were based on respondents’ self-reports. To avoid bias, further studies should consider clinical assessment for depressive symptoms and more accurate and objective evaluation of caregiving hours. Finally, we were unable to obtain detailed information about care recipients and caregiving situations. For instance, spousal or cohabitant caregivers [3, 39] and family caregivers for individuals with dementia [15, 16] have an increased risk for higher caregiving burden and health decline. These characteristics could confound the findings.

### Implications

Despite the limitations, this study adds novel findings to the existing literature on caregiver burden and its health consequences by providing evidence for income inequalities even under an LTCI system. Policymakers and professionals working with older adults and their families should dedicate more focus to family caregiver burden and its consequence of health decline, and consider methods for decreasing income-based inequality. This study was conducted in Japan, which has implemented the LTCI system for more than a decade; however, traditional familism could still affect family caregiving [20]. Since health disparities in caregivers could be inconsistent according to welfare regimes [40] and ethnicities [37], more comparative studies are necessary to examine health disparities in caregiver populations.

Future studies in Japan should also examine in detail the causes of long caregiving hours observed in low-income households, such as service utilization patterns and specific aspects of care provided. In 2015, in Japan, the LTCI system revised co-insurance for service users, from a singular ratio of 10%, to a relative ratio of 10–20%, contingent upon the income level of older adults, which will be revised to 10–30% from 2018. At the same time, the LTCI system reduced the monthly care premium for older adults with lower income [21]. That said, since our study findings were based on a survey conducted in data prior to these changes, a follow-up to examine the potential effects of these revisions is important.

Regarding disparity in depressive symptoms, our findings suggest necessity for comprehensive efforts to reduce it for whole older population beyond the LTCI system. On the other hand, support programs should be considered in the LTCI system for alleviating depressive symptoms in family caregivers regardless of income levels. Caregiver burden is a leading cause for institutionalization [41, 42]. Despite the Japanese LTCI system aiming to promote home- and community-based rather than institutional care, it does not regulate services for family caregivers themselves to alleviate their burden or health decline caused by caregiving. To alleviate the excess risk for depressive symptoms, support for caregivers themselves, such as caregiver assessment [43] and evidence-based support programs [44, 45] should be implemented.

## Conclusion

Using data obtained from a nationwide survey, this study examined income-based inequalities in older family caregivers under an LTCI system. Our findings showed the disparity in caregiving hours and similar, but not larger, inequalities in depressive symptoms in caregivers compared to non-caregivers. These findings suggest that policymakers and professionals within the LTCI system should consider such disparity; at the same time, more support for alleviating caregivers’ depressive symptoms should be implemented regardless of their income, rather than providing exclusive support for caregivers with a low income.

## Supporting information

S1 Table Characteristics of caregivers according to engagement in long caregiving hours (≥72). (PDF)

S2 Table Characteristics of non-caregivers according to geriatric depression scale score (≥5). (PDF)
