# Supplementary material for: Income-based inequalities in caregiving time and depressive symptoms among older family caregivers under the Japanese long-term care insurance system: A cross-sectional analysis
Source: PLoS One. 2018 Mar 28;13(3):e0194919. doi: 10.1371/journal.pone.0194919 (PMC5874058; doi:10.1371/journal.pone.0194919)
Supplement: S1 Table — (PDF) [file pone.0194919.s001.pdf]

S1 Table. Characteristics of Caregivers According to Engagement in Long Caregiving Hours (≥72)

**Variables**	**<72 hours (*n* = 1,285) *M* (SD)[a] or %**	**≥72 hours (*n* = 313) *M* (SD)[a] or %**	***p*[b]**
**Age (range = 65–98 years)**	72.8 (6.1)	74.3 (6.0)	< .001
**Gender (Male)**	47.5	29.7	< .001
**Income**			.001
1st quartile	20.6	10.9	
2nd quartile	24.0	22.7	
3rd quartile	19.6	22.7	
4th quartile	23.0	26.8	
Public assistance	0.9	1.9	
Missing	11.9	15.0	
**Education**			.012
<10 years	32.3	40.6	
≥10 years	66.4	57.5	
Missing	1.3	1.9	
**Marital status**			.049
Married	85.8	89.8	
Not married	11.4	6.7	
Missing	2.9	3.5	
**Work engagement**			< .001
Yes	22.3	9.9	
No	73.9	82.7	
Missing	3.8	7.3	
**Presence of disease**			.439
None	15.5	12.1	
One	32.3	34.8	
Two or more	46.4	46.3	
Missing	5.8	6.7	

[a]M: mean; SD: standard deviation.

[b]Differences in distributions between caregivers with or without engagement in ≥72 caregiving hours were assessed using chi-squared tests. Difference in mean age was compared using a t-test.
