# Supplementary material for: Income-based inequalities in caregiving time and depressive symptoms among older family caregivers under the Japanese long-term care insurance system: A cross-sectional analysis
Source: PLoS One. 2018 Mar 28;13(3):e0194919. doi: 10.1371/journal.pone.0194919 (PMC5874058; doi:10.1371/journal.pone.0194919)
Supplement: S2 Table — (PDF) [file pone.0194919.s002.pdf]

**S2 Table. Characteristics of Non-Caregivers According to Geriatric Depression Scale Score (≥5)**

| **Variables** | **GDS[a] (<5) (*n* = 12,469) *M* (SD)[b] or %** | **GDS[a] (≥5) (*n* = 4,172) *M* (SD)[b] or %** | ***p*[c]** |
|---|---|---|---|
| **Age (range = 65–98 years)** | 73.4 (5.9) | 74.3 (6.4) | < .001 |
| **Gender (Male)** | 49.7 | 50.3 | .531 |
| **Income** | | | < .001 |
| 1st quartile | 24.0 | 11.8 | |
| 2nd quartile | 24.4 | 20.1 | |
| 3rd quartile | 20.3 | 21.2 | |
| 4th quartile | 17.9 | 30.2 | |
| Public assistance | 1.0 | 2.7 | |
| Missing | 12.4 | 13.9 | |
| **Education** | | | < .001 |
| <10 years | 36.5 | 47.0 | |
| ≥10 years | 62.5 | 51.5 | |
| Missing | 1.1 | 1.5 | |
| **Marital status** | | | < .001 |
| Married | 74.9 | 65.7 | |
| Not married | 23.6 | 31.3 | |
| Missing | 1.5 | 3.0 | |
| **Work engagement** | | | < .001 |
| Yes | 25.8 | 18.1 | |
| No | 70.2 | 76.8 | |
| Missing | 4.0 | 5.1 | |
| **Presence of disease** | | | < .001 |
| None | 17.3 | 9.6 | |
| One | 36.4 | 30.9 | |
| Two or more | 39.6 | 54.8 | |
| Missing | 6.8 | 4.7 | |

[a]GDS: Geriatric Depression Scale

[b]M: mean; SD: standard deviation

[c]Differences in distributions between caregivers with or without GDS (≥5) were assessed using chi-squared tests. Difference in mean age was compared using a t-test.
